# Supplementary material for: Distinct transcriptional remodeling of immune and metabolic pathways in severe undernutrition during latent tuberculosis infection does not consistently predict tuberculosis progression across independent cohorts
Source: bioRxiv. 2026 Oct 28:2026.10.26.756475. Preprint. [Version 1] doi: 10.64898/2026.10.26.756475 (PMC13733851; doi:10.64898/2026.10.26.756475)
Supplement: Supplementary file 1 [file media-1.pdf]

Supplementary Material

Supplementary Methods, Tables S1-S6 and Supplementary Data S1 manifest

Supplementary Methods

Datasets and prespecified analytical structure

These supplementary resources report the exact R-confirmed outputs supporting the staged secondary analysis. GSE152218 provided the severe-undernutrition discovery contrast in adults with latent tuberculosis infection (LTBI) and the exploratory internal active-TB gate. GSE79362 provided prospective programme projection, and GSE94438/GC6 provided outcome-independent assay-portability-gated projection. The external cohorts test transportability of the discovery-defined programme rather than direct replication of the BMI-defined nutritional contrast.

Discovery cohort definition and sensitivity analysis

Adults with LTBI in GSE152218 were selected when BMI, age and sex metadata were available. Severe undernutrition was defined as BMI <16 kg/m2 and adequate nutritional status as BMI >=18.5 kg/m2. The complete model included 16 severely undernourished and 11 adequately nourished participants. Sample 102-00459-B was retained in the complete model and omitted in the prespecified sensitivity model following quality-control review.

Differential-expression modelling and Reactome enrichment

Gene-level modelling used trimmed-mean-of-M-values-normalised counts and limma-voom precision-weighted linear models adjusted for age and sex. Reactome enrichment used fgseaMultilevel on moderated t-statistic rankings after Ensembl-to-symbol harmonisation. A stable pathway required concordant direction and false discovery rate <0.05 in both complete and sensitivity models. Seven representative modules were locked before external outcome testing.

Internal active-TB convergence gate

The internal reference axis compared adults with severe undernutrition and active TB with adults with severe undernutrition and LTBI. Gene-level log2-fold-change and moderated-t convergence and pathway-level normalised enrichment-score convergence were assessed. This gate is interpreted as exploratory because of covariate imbalance between disease-state groups.

Prospective projection and assay portability

All seven exact-confirmed modules were scoreable in GSE79362. In GC6, an outcome-independent portability rule was applied before outcome testing: a module was retained only if the GC6 curated assay represented at least 60% of genes represented in GSE79362 and included at least 20 genes. Consequently, only four technically portable modules were evaluated in GC6 outcome analyses.

Reproducibility archive

Supplementary Data S1 supplies the exact machine-readable output tables, frozen module definition file, metadata audits, R scripts and session-information files supporting the numerical findings reported in the article and in Tables S1-S6.

Supplementary Tables

Table S1. Discovery cohort sample audit for the GSE152218 adult LTBI nutritional-status comparison.

GEO sample	Participant ID	Age	Sex	BMI (kg/m2)	Severe-undernutrition group	Included
GSM4609386	102-00042-B	45	female	14.81	Severe undernutrition (<16)	Yes
GSM4609387	102-00088-B	23	male	13.92	Severe undernutrition (<16)	Yes

GEO sample	Participant ID	Age	Sex	BMI (kg/m2)	Severe-undernutrition group	Included
GSM4609388	102-00129-B0	25	male	24.15	Not severe (>=16)	Yes
GSM4609389	102-00131-B	40	female	15.09	Severe undernutrition (<16)	Yes
GSM4609390	102-00171-B	39	female	28.12	Not severe (>=16)	Yes
GSM4609391	102-00188-B	21	female	22.87	Not severe (>=16)	Yes
GSM4609393	102-00217-B	40	female	27.81	Not severe (>=16)	Yes
GSM4609395	102-00240-B	50	female	27.44	Not severe (>=16)	Yes
GSM4609396	102-00241-B	19	female	13.87	Severe undernutrition (<16)	Yes
GSM4609397	102-00246-B	34	female	15.61	Severe undernutrition (<16)	Yes
GSM4609400	102-00265-B0	24	female	14.21	Severe undernutrition (<16)	Yes
GSM4609401	102-00275-B	46	male	33.16	Not severe (>=16)	Yes
GSM4609402	102-00277-B	22	male	19.52	Not severe (>=16)	Yes
GSM4609404	102-00291-B	23	female	21.01	Not severe (>=16)	Yes
GSM4609405	102-00298-B	22	female	30.83	Not severe (>=16)	Yes
GSM4609407	102-00304-B	60	male	19.09	Not severe (>=16)	Yes
GSM4609409	102-00308-B0	37	female	26.31	Not severe (>=16)	Yes
GSM4609410	102-00310-B	18	male	15.68	Severe undernutrition (<16)	Yes
GSM4609413	102-00323-B	20	male	15.72	Severe undernutrition (<16)	Yes
GSM4609421	102-00350-B	36	female	15.66	Severe undernutrition (<16)	Yes
GSM4609423	102-00365-B	46	female	14.38	Severe undernutrition (<16)	Yes
GSM4609424	102-00373-B	54	male	13.72	Severe undernutrition (<16)	Yes
GSM4609426	102-00374-B0	22	female	14.04	Severe undernutrition (<16)	Yes
GSM4609430	102-00459-B	19	female	15.82	Severe undernutrition (<16)	Yes
GSM4609431	102-00467-B	57	female	15.81	Severe undernutrition (<16)	Yes
GSM4609432	102-00525-B	31	male	15.17	Severe undernutrition (<16)	Yes
GSM4609433	102-00548-B	30	male	15.81	Severe undernutrition (<16)	Yes

Table S2. Canonical discovery and sensitivity-analysis robustness summary.

Output type	Metric/model	Samples	Genes tested	Value / FDR<0.05	FDR<0.10
Differential-expression model	all_27_samples	27	20382	0	0
Differential-expression model	sensitivity_excluding_102-00459-B	26	20059	2	9
Gene-level robustness	Spearman correlation of log2FC	-	-	0.928305	-
Gene-level robustness	Direction agreement	-	-	0.906974	-
Gene-level robustness	Shared genes at FDR <0.05	-	-	0	-
Reactome robustness	Full model pathways at FDR <0.05	-	-	311	-
Reactome robustness	Sensitivity model pathways at FDR <0.05	-	-	264	-
Reactome robustness	Stable concordant pathways at FDR <0.05	-	-	241	-
Reactome robustness	Stable concordant pathways at FDR <0.10	-	-	293	-
Reactome robustness	Pathway NES Spearman correlation	-	-	0.95446	-
Reactome robustness	Pathway direction agreement	-	-	0.898903	-

Table S3. Exact-confirmed seven-module programme used for prospective projection.

Theme	Representative pathway	Locked direction	Full NES	Full FDR	Sensitivity NES	Sensitivity FDR
Complement / Fc-receptor effector activity	Classical antibody-mediated complement activation	Higher in severe undernutrition	3.555	5.79e-31	3.567	2.57e-32
Neutrophil / innate granule programmes	Neutrophil degranulation	Lower in severe undernutrition	-2.788	3.03e-32	-2.654	1.41e-28
Metabolism / mitochondrial energetics	Role of phospholipids in phagocytosis	Higher in severe undernutrition	3.413	2.30e-26	3.336	5.80e-28
Antigen presentation / adaptive immunity	CD22 mediated BCR regulation	Higher in severe undernutrition	3.485	4.21e-27	3.488	2.48e-28
Haemostasis / platelet / vascular	Cell surface interactions at the vascular wall	Higher in severe undernutrition	2.864	1.78e-21	2.830	4.45e-19
Stress / cell death / repair	Autophagy	Lower in severe undernutrition	-2.087	3.05e-06	-2.171	1.90e-07
Interferon / cytokine signalling	TNFR2 non-canonical NF-kB pathway	Lower in severe undernutrition	-1.953	9.54e-04	-1.907	4.72e-04

Table S4. Internal active-TB convergence-gate summary.

Evidence level	Metric	Value
Gene-level	Shared genes	19787
Gene-level	Spearman correlation of logFC	-0.398962
Gene-level	Spearman correlation of moderated t	-0.42693
Gene-level	Directional agreement	0.349371
Pathway-level	Common Reactome pathways	1274
Pathway-level	Pathway NES Spearman correlation	-0.390255
Pathway-level	Pathway direction agreement	0.342229
Pathway-level	Exact stable undernutrition pathways evaluated	241
Pathway-level	Exact stable pathways same direction	63
Pathway-level	Exact stable pathways opposite direction	178
Pathway-level	Opposite-direction stable pathways with activeTB FDR <0.05	117

Interpretation boundary: this comparison is an exploratory mechanistic gate rather than independent clinical validation.

Table S5. GSE79362 programme representation and prospective projection outcomes.

Module theme	Represented genes	Coverage	Projection status
Complement / Fc-receptor effector activity	73	83.0%	Scoreable
Neutrophil / innate granule programmes	427	89.3%	Scoreable
Metabolism / mitochondrial energetics	92	86.0%	Scoreable
Antigen presentation / adaptive immunity	59	84.3%	Scoreable
Haemostasis / platelet / vascular	159	68.2%	Scoreable
Stress / cell death / repair	130	81.2%	Scoreable
Interferon / cytokine signalling	74	84.1%	Scoreable

Analysis	Progressors/paired n	Controls	Median difference/change	Unadjusted P	Adjusted OR/SD	Adjusted P
Baseline: future progression at any follow-up	28	100	-0.0205	0.5056	1.090	0.6922
Baseline: TB progression within 12 months	9	100	-0.0227	0.8645	-	-
Paired prediagnostic trajectory	19	-	0.0494	0.0671	-	-

Table S6. GC6 assay-portability gate and technically gated outcome analyses.

Module theme	GSE79362 genes	GC6 genes	Retention	Portability decision
Complement / Fc-receptor effector activity	73	5	6.8%	Excluded
Neutrophil / innate granule programmes	427	442	103.5%	Retained
Metabolism / mitochondrial energetics	92	23	25.0%	Excluded
Antigen presentation / adaptive immunity	59	5	8.5%	Excluded
Haemostasis / platelet / vascular	159	114	71.7%	Retained
Stress / cell death / repair	130	142	109.2%	Retained
Interferon / cytokine signalling	74	82	110.8%	Retained

Analysis	Progressors/paired n	Controls	Median difference/change	Unadjusted P	Adjusted OR/SD	Adjusted P
Month 0 baseline: any future TB progression	64	208	-0.0524	0.0220	0.711	0.0218
Month 0 baseline: progression within 12 months	38	208	-0.0683	0.0325	0.710	0.0588
Month 6 landmark: TB within next 12 months	14	64	0.0393	0.5363	0.711	0.2851
Paired prediagnostic trajectory	20	-	-0.0977	0.2549	-	-

Supplementary Data S1. Exact-output and reproducibility archive

Supplementary Data S1 is provided as a ZIP archive containing exact R-confirmed output tables, frozen module definitions, metadata audit files, R scripts and R session-information files. A SHA-256 checksum manifest is included within the archive.
