# Supplementary material for: Distinct transcriptional remodeling of immune and metabolic pathways in severe undernutrition during latent tuberculosis infection does not consistently predict tuberculosis progression across independent cohorts
Source: bioRxiv. 2026 Oct 28:2026.10.26.756475. Preprint. [Version 1] doi: 10.64898/2026.10.26.756475 (PMC13733851; doi:10.64898/2026.10.26.756475)
Supplement: Supplementary file 2 [file media-2.pdf]

Supplementary Tables S7-S10

Distinct transcriptional remodeling of immune and metabolic pathways in severe undernutrition during latent tuberculosis infection does not consistently predict tuberculosis progression across independent cohorts

Supplementary Table S7. Public datasets, study populations and prespecified analytical roles.

Dataset	Population and setting	Analytical use	Key design safeguard
GSE152218	Adults with LTBI or active TB; India; whole-blood RNA-seq	Discovery contrast and exploratory active-TB comparison	Nutrition discovery restricted to adult LTBI; active-TB comparison treated as exploratory
GSE79362	South African adolescents with M. tuberculosis infection followed longitudinally	Prospective seven-module score testing	Baseline primary analysis; paired trajectory secondary analysis
GSE94438 / GC6	African household contacts sampled longitudinally after exposure	Progression testing after pathway-representation assessment	Outcome-independent pathway-representation criterion before scoring

Supplementary Table S8. Representative undernutrition-associated pathways and GC6 assay-representation status.

Biological theme	Representative Reactome pathway	Direction in severe undernutrition	Sensitivity NES	Sensitivity FDR	GC6 representation criterion
Complement / Fc-receptor effector activity	Classical antibody-mediated complement activation	Higher in severe undernutrition	3.567	2.57e-32	Did not meet
Neutrophil / innate granule programs	Neutrophil degranulation	Lower in severe undernutrition	-2.654	1.41e-28	Met
Phospholipid metabolism / phagocytosis	Role of phospholipids in phagocytosis	Higher in severe undernutrition	3.336	5.80e-28	Did not meet
Antigen presentation / adaptive immunity	CD22 mediated BCR regulation	Higher in severe undernutrition	3.488	2.48e-28	Did not meet
Hemostasis / platelet / vascular	Cell surface interactions at the vascular wall	Higher in severe undernutrition	2.830	4.45e-19	Met
Stress / cell death / repair	Autophagy	Lower in severe undernutrition	-2.171	1.90e-07	Met
Interferon / cytokine signaling	TNFR2 non-canonical NF-kB pathway	Lower in severe undernutrition	-1.907	4.72e-04	Met

Supplementary Table S9. Major analytical questions and interpretation boundaries.

Analytical question	Exact result	Interpretation
Is severe undernutrition associated with differences in LTBI blood transcriptional biology?	241 stable Reactome pathways; 7/7 representative pathways directionally confirmed in both models	Yes, at the coordinated pathway level
Does it yield a robust standalone gene biomarker signature?	0 FDR-significant genes in the full discovery model	No
Does the undernutrition-associated pattern resemble active TB?	Pathway NES rho=-0.390; 178/241 stable pathways opposite	No; predominantly inverse in the exploratory internal comparison
Does it predict progression in GSE79362 at baseline?	Adjusted OR per SD=1.090; P=0.6922	No evidence
Can all seven predefined modules be evaluated in GC6 under the prespecified representation criterion?	4/7 modules meet the pathway-representation criterion	No; the full seven-module score cannot be evaluated in GC6
Do the four evaluable GC6 modules associate with progression at baseline?	Adjusted OR per SD=0.711; P=0.0218; robust to shared-gene and strict-overlap sensitivity analyses	Inverse baseline association; not consistently confirmed in secondary or longitudinal analyses

Supplementary Table S10. Post hoc GC6 gene-identity overlap audit and score-sensitivity analyses.

A. Gene-identity overlap for the seven predefined pathway modules.

Module	GSE79362 genes	GC6 genes	Shared genes	Shared / GSE79362 (%)	Jaccard (%)	Prespecified count-ratio criterion	Strict identity-overlap criterion
Complement / Fc-receptor effector activity	73	5	4	5.5	5.4	Did not meet	Did not meet
Neutrophil / innate granule	427	442	421	98.6	94.0	Met	Met
Phospholipid metabolism / phagocytosis	92	23	23	25.0	25.0	Did not meet	Did not meet
Antigen presentation / adaptive immunity	59	5	5	8.5	8.5	Did not meet	Did not meet
Hemostasis / platelet / vascular	159	114	91	57.2	50.0	Met	Did not meet
Stress / cell death / repair	130	142	130	100.0	91.5	Met	Met
Interferon / cytokine signaling	74	82	72	97.3	85.7	Met	Met

B. GC6 outcome sensitivity across alternative cross-assay score definitions.

Analysis	Score definition	Cases / controls	Adjusted OR per SD	Adjusted P	Median difference / paired delta	Unadjusted P
Month 0: any future TB	Current 4-module	64 / 208	0.711	0.02178	-0.0524	0.02199
Month 0: any future TB	Shared-gene 4-module	64 / 208	0.713	0.02236	-0.0553	0.01988
Month 0: any future TB	Strict-overlap 3-module	64 / 208	0.696	0.01445	-0.0768	0.01508
Month 0: TB within 12 months	Current 4-module	38 / 208	0.710	0.05884	-0.0683	0.03249
Month 0: TB within 12 months	Shared-gene 4-module	38 / 208	0.707	0.05454	-0.0743	0.03034
Month 0: TB within 12 months	Strict-overlap 3-module	38 / 208	0.708	0.05416	-0.0856	0.03310
Month 6 landmark	Current 4-module	14 / 64	0.711	0.28510	+0.0393	0.53630
Month 6 landmark	Shared-gene 4-module	14 / 64	0.682	0.23010	+0.0370	0.43090
Month 6 landmark	Strict-overlap 3-module	14 / 64	0.600	0.11760	+0.0101	0.37950
Paired prediagnostic	Current 4-module	20 paired	-	-	-0.0977	0.2549
Paired prediagnostic	Shared-gene 4-module	20 paired	-	-	-0.1017	0.1978
Paired prediagnostic	Strict-overlap 3-module	20 paired	-	-	-0.1196	0.2250

Notes: The prespecified GC6 criterion compared pathway gene counts across assays: GC6 had to contain at least 60% as many measurable pathway genes as GSE79362 and at least 20 pathway genes. The shared-gene four-module sensitivity recalculated the same four modules using only genes measurable in both datasets. The strict-overlap three-module sensitivity additionally required at least 60% of the GSE79362-represented genes to be shared with GC6 and at least 20 shared genes; this excluded the hemostasis/vascular module. These analyses were post hoc robustness checks and do not replace the prespecified four-module analysis.
